# Data Note: Social role transitions (further/higher education, employment, living situation, parenthood, and being a carer) in the G1s of the Avon Longitudinal Study of Parents and Children (ALSPAC)

**DOI:** 10.12688/wellcomeopenres.23282.2

**Published:** 2025-11-17

**Authors:** Annie Herbert, Ingrid Schoon, Anne McMunn, Rebecca Lacey, Jon Heron, Laura Howe

**Affiliations:** 1 MRC Integrative Epidemiology Unit, Bristol, England, UK; 2 Population Health Sciences, University of Bristol, Bristol, UK; 3 Centre for Academic Mental Health, University of Bristol, Bristol, UK; 4 University College London Social Research Institute, London, England, UK; 5 International Centre for Life Course Studies in Society and Health, University College London, London, UK; 6 University College London Research Department of Epidemiology and Public Health, London, England, UK; 7 St George's University of London Population Health Research Institute, London, England, UK

**Keywords:** Social inequalities, Work and family, Life-course, Transition to adulthood, Avon Longitudinal Study of Parents and Children, Prospective cohort, United Kingdom

## Abstract

**Background:**

Social roles common to adulthood (e.g. employment, parenthood) and their timing and combinations have been shown to relate to health. However, there is a lack of contemporary data to study complex patterns. We applied a pragmatic algorithmic approach to data from the ALSPAC (Avon Longitudinal Study of Parents and Children) G1 cohort born in 1991–1993 to build valid annual indicators at age 16–31 of being in (or out of) six key roles.

**Methods:**

In February 2023 we searched the data catalogue and identified 449 relevant variables (318 root questions, 131 auxiliary) indicating if an individual had been or was currently in the following roles: further/higher education; employment; living away from the parental home; cohabiting with a partner/spouse; parenthood; being a carer. Variables were incorporated into four algorithms to determine whether each individual was in/out of a role per age year. We addressed missing indicator values with multiple imputation methods. We assessed how well indicators captured annual role status by using them to derive descriptive statistics and comparing with those from national and survey data from the same period.

**Results:**

Descriptive statistics on transitioning to or leaving a particular role by age 30 were comparable to national official data. For example, by age 30, at least 27% of men and 50% of women indicated having left the parental home (at median age 23–24); of these individuals, 19–30% subsequently indicated living with their parents again by age 30 (median interval 2–3 years, interquartile range 1–4 years). However, employment and parenthood appeared to be under-captured, relative to the other four roles.

**Conclusions:**

These indicators can now be used by other researchers, for example to study a particular role or construct (e.g. Not in Education, Employment, or Training) over time, in different social and health contexts. They are best used to derive composite variables for use in life course research, rather than for precise year-on-year sequence analysis and parenthood should be used with caution

## Background

Young adulthood is a critical developmental period in the life-course ^1– 3^. Transitions to new social roles (leaving full-time education, starting training/employment; leaving the family home; cohabitation; marriage; parenthood) that occur during this time, have been shown to influence health and related behaviours ^4– 9^. For example, early or clustered social roles, or relatively late or few social roles, are associated with worse health-related outcomes, such as steeper trajectories of body mass index ^5^, increased likelihood of smoking ^6^, and decreased life satisfaction ^10^.

There is a lack of evidence on social roles in today’s young people. Work in UK cohorts born in the 1950s to 1970s indicate that patterns of social roles are becoming more complex in later-born cohorts ^11– 13^, which may be even more complex in today’s young adults (born in the 1990s and 2000s). This includes a lack of consideration of transitions being ‘reversed’ (e.g. moving back into the family home or leaving employment), despite such events being potentially very damaging to health ^14^. This lack is likely due to a scarcity of nuanced data to support such work ^8, 15, 16^. Contemporary data sources that allow for a better understanding of longitudinal patterns of social roles, including the stability of these roles, is crucial, given today’s unique pressures on young people, such as changing access to higher education, housing, job markets, and long-term healthcare ^3, 17– 21^. Such understanding would also be timely given that the UK Government and public bodies are looking for ways to better support young people, support that is likely to have downstream effects on health ^22, 23^.

Finally, there is increasing recognition that many young adults are informal/unpaid (non-parental) ‘carers’ (around 1 in 10 in Europe) ^24^. The majority provide day-to-day care for a parent or grandparent with a long-term health condition ^25^, for a median of 20–49 hours a week ^26^. Assuming the role of carer at a relative early age has been shown to impact a young person’s participation in other adult social roles, as well as impact on their health ^27^, and is likely becoming more common over time given ageing populations and longer life expectancy. Therefore, its consideration when researching patterns of young adult social roles – which until now has been little – is warranted ^8^. With the above in mind, we took advantage of the rich longitudinal data in the Avon Longitudinal Study of Parents and Children (ALSPAC) UK 1990’s-born cohort. We used a pragmatic and algorithmic approach to draw out information from ~400 variables capturing presence/absence and timing of social roles in the G1s (children of the ALSPAC pregnancies), to create annual indicators of six social roles from age 16 until 30, including being a young adult carer.

The primary motivation for developing these annual social role indicators was to study different patterns of transitions into and out of these roles at ages 18–25, as part of a project focussing on the potential causal impact of different patterns for subsequent health. However, we have broadened the scope of how these variables are derived, the age range for the indicators up to the latest possible time-point (age 31), and made R programming scripts freely available and flexible, such that:

a) these indicators can be taken forward and used for broader social research using ALSPAC (e.g. modifying the definition of each role to suit the user, or studying other outcomes);

b) indicators can be created for later age years as new waves of ALSPAC data become available.

In this Data Note, we describe how the indicators are derived; validation of these indicators through comparing descriptive statistics to official national statistics and other evidence sources; how data can be accessed, and scripts updated to suit future research needs.

## Methods

### The ALSPAC cohort

ALSPAC (Avon Longitudinal Study of Parents and Children, also known as ‘Children of the 90s’), is a UK birth cohort of ~14,500 mothers pregnant in the Avon area, with expected delivery dates in April 1991–December 1992 (approximately three-quarters of the eligible population were recruited). Initially 14,541 pregnancies were enrolled; 13,988 children were alive at 1 year of age. When the oldest children were approximately 7 years of age, an attempt was made to bolster the initial sample with eligible cases who had failed to join the study originally, resulting in an additional 913 children being enrolled. The total sample size for analyses using any data collected after the age of seven is therefore 15,447 pregnancies; 14,901 children were alive at 1 year of age. Information has been regularly collected since enrolment until the present. Study data were collected and managed using REDCap electronic data capture tools hosted at the University of Bristol ^28^. More information on ALSPAC data is available at the study website ( http://www.bristol.ac.uk/alspac/), with a fully searchable data dictionary and variable search tool, and in cohort profiles about the recruited mothers and their offspring, respectively ^29– 31^. Ethical approval for the study was obtained from the ALSPAC Ethics and Law Committee and the Local Research Ethics Committees.

To capture the most recent young adults, we focussed on the ‘G1s’, the children from the pregnancies in recruitment, who were age 32–34 at the time of writing (G0s being the parents of these pregnancies). As there are likely to be different inter-relationships between social role variables, by gender, we aimed to derive social role indicators in the 15,601 G1s who had non-missing data on sex (in ALSPAC gender identity is not available, so is approximated by biological sex at birth). When referring to the age ‘X’ survey, this is the age at which it was intended for the G1 to be surveyed. Given the large cohort, and delays in responding to surveys or invitations to clinics, participants could be surveyed in the year leading up to their Xth birthday, and within a year or two after. The median ages at specific sweeps are provided in Extended Data File 1 ^32, 33^, which provides meta-data about all the variables used in this project.

We note here that the G1s would have been age 16–17 in 2006–2009, as this has implications for decisions made about defining further/higher education, employment, and leaving the parental home statuses, below.

### Variables used to capture social roles

We searched the ALSPAC data catalogue in February 2023 and identified 318 root questions where responses indicated whether an individual had been, or was currently, in the following social roles, respectively: further or higher education (non-mandatory post-age 18 college/university); employment (30+ hours or equivalent vocational training); left parental home (including de-facto guardians); cohabiting with a partner/spouse; become a parent; become a carer. Root questions were asked both in surveys (postal and online) and within clinics (computer-assisted surveys) from ages 16 to 31. Questions could either:

a) directly enquire about that social role, e.g. at the age 18 survey: ‘Do you live with any of the following people? If you are a student please answer the question about the people you live with during term time. (For each of these statements listed below please cross one box to indicate whether or not this applies to you.)’ – ALSPAC variable ccu4039, directly capturing whether living with parents, or cohabiting with a partner;

b) indirectly (unintentionally) capture a social role status, e.g. for the same question/ALSPAC variable ccu4039 asking about living status, a response option was ‘lives in halls of residence’ – we used this response to determine that the individual was in further/higher education, even though the original question had not intended to capture student status. For these questions, responses were sufficient to indicate that an individual was in that role, but a lack of this response was not sufficient to indicate not being in that role. In the previous example, not living in halls of residence was not enough to mean that the individual was not in further/higher education. Note that the same indirect question could be used to inform indicators at the same age-point, across different social roles (e.g. living in halls of residence indicated that an individual was both in further/higher education and did not live with their parents).

An additional 131 variables provided relevant auxiliary information about roles which could be used to either further help determine if someone was in a particular role (e.g. root variable ype9930 indicated that an individual lived with Person A; auxiliary variable ype9910 indicated that Person A was the individual’s partner), or timing of the role (e.g. root variable ccu4055 indicates an individual’s current main educational or training activity; auxiliary variable ccu9991 provides the exact age at the time of responding to the questionnaire that included the question capturing variable ccu4055).

All root and auxiliary questions are listed in Extended Data File 1, with descriptive data in terms of numbers and types of questions provided in Table 1. The responses to these questions were eventually used to derive binary indicators for each role (0=currently not in role, 1=currently in role), at each year of age.

**Table 1.** Descriptive statistics about question variables used to derive social role indicators in ALSPAC G1s.

Role	Number of root question variables available	Number of root question variables after exclusions *, N	n (% of N) ‘current’ variables		n (% of N) ‘date’ variables		n (% of N) ‘since’ variables		n (% of N) ‘age’ variables		n (% of N) with information on exact age at time of questioning **		n (% of N) with information on exact date transition first took place **	
Further/higher education	37	32	23	(72)	1	(3)	8	(25)	0	(0)	31	(97)	2	(6)
Higher education	14	13	6	(46)	1	(8)	6	(46)	0	(0)	12	(92)	1	(8)
Employment (any)	101	99	68	(69)	6	(6)	24	(24)	1	(1)	99	(100)	16	(16)
Full-time employment	38	38	38	(100)	0	(0)	0	(0)	0	(0)	38	(100)	4	(11)
Left parental home	26	26	12	(46)	0	(0)	13	(50)	1	(4)	26	(100)	0	(0)
Cohabiting	45	28	19	(68)	0	(0)	9	(32)	0	(0)	28	(100)	0	(0)
Parenthood	120	104	79	(76)	0	(0)	25	(24)	0	(0)	103	(99)	15	(14)
Carer	7	7	7	(100)	0	(0)	0	(0)	0	(0)	7	(100)	0	(0)

‘current’, ‘date’, ‘since’, and ‘age’, refer to different algorithms used, described under ‘Deriving annual indicators’. Note that some questions can apply to more than one role. *Specific to the authors’ original project/exclusion criteria specified under ‘Social role variables’. **To nearest month.

### Definitions of social roles

We defined roles as described below. We defined roles to represent activity that would pass a certain threshold of social status, time occupation/management, responsibility, or change in levels of support, that could feasibly have an impact on later health ^34, 35^. Leaving the parental home and parenthood was captured from age 16, employment from age 17; further/higher education from age 18; cohabitation from age 20; being a carer from age 22.

**Further/higher education** was defined as any education after secondary school at college or university, and the person’s main activity. In the UK in 2006–2009, the earliest possible legal school leaving age was 16 (it is currently 18); we do not include any questions mentioning education at ages 16–17 as these 16–17 year olds could still be in secondary education, and entry to university rarely occurs before the age of 18. We included questions that mentioned ‘full-time education’, ‘studying for qualifications’, ‘studying for a degree’, or ‘university’, and captured status after age 18, assuming this to mean either further or higher education. Note that this means that we can separate out higher education (‘studying for a degree’ or ‘university’), but not further education – this was an additional reason for not including instances of further education at ages 16–17. Post-age 18 questions that mentioned ‘educational activities’ or ‘part-time education’ without any indication of it being the person’s main activity were not included – these questions were considered to not be specific enough and possibly included recreational educational activities.

We derived indicators for both any employment, and specifically full-time employment. **Employment** was defined as any employment (including self-employment) or vocational training, as we considered vocational training to involve activities more closely matching the employment role than further/higher education role. Employment was considered to be **full-time** when it was the person’s main activity, where ‘main activity’ is assumed to equate to at least 30 hours work a week ^36^. Questions stating ‘irregular hours’ or ‘gig-economy’ work roles were included under ‘any employment’ but excluded from the definition of full-time working, as hours worked were never specified, and it was highly possible that these roles involved <30 hours work per week ^37^. When asked about ‘main activity’ the response ‘Stay At Home Parent’ was assumed to mean that they were not employed. In England in 2006–2009, those aged 16–17 were considered ‘young workers’ ^38^, with tighter restrictions regarding main activities compared to those aged 18+ (though full-time working and vocational training were possible). Our list of root questions includes only one employment question before age 18 – one at age 17 specifically capturing whether the young person worked 30+ hours a week or was in a ‘modern apprenticeship’. A small proportion of the employment questions (17/113) specified if the work was paid.

**Left parental home** was defined as living with no parents (including de-facto guardians), regardless of who else lived in the home (e.g. a partner). We hypothesised that the presence of parents themselves, regardless of others in the home, can affect an individual’s health – for example, through increased resources, support, advice, parental monitoring, or reinforcement of healthy behaviours, or the status of living with parents based on socio-cultural norms, impacting a young person’s self-identity and therefore their mental health, both positively and negatively ^16, 34, 39^. In 2006–2009, the earliest possible legal age to leave the parental (guardian) home was 16 with the parent’s/guardian’s permission, 18 without it.

**Cohabiting** was defined as living with a romantic/intimate partner (referred to in questions as ‘partner’, ‘spouse’, ‘girlfriend’ or ‘boyfriend’). An individual was cohabiting if they were living with their intimate partner, regardless of whether they were still in the parental home (e.g. the partner had moved into their parents’ home). We hypothesised that sharing one’s daily lives with an intimate partner could affect health independent of whether living with parents, for example through similar (but separate) mechanisms of increased resources/support/advice/reinforcement of healthy behaviours ^40, 41^. To better capture as many instances of cohabiting as possible, we included questions that indicated that an individual had a spouse/civil partnership (even if it did not specify that the person lived with that spouse) – based on marriage passing a certain threshold of commitment in a relationship, and that official statistics indicate that for married couples born in the UK in recent decades, approximately only 3% live apart ^42^. For responses that indicated that an individual lived with their partner’s mother or father, we assumed that they also lived with their partner (i.e. that they were cohabiting).

**Parenthood** was defined as becoming a parent or de-facto guardian of a live-born child. Within the context of considering social roles in young adulthood, our original research question that motivated how we defined social roles viewed the impact of parenthood on health through the mental load and physical labour that comes with being day-to-day responsible for a child ^43^. Therefore, we included responses to questions indicating that the individual was de-facto parent and not necessarily a biological parent (e.g. had step, foster, or adopted children). We took responses that a child had died, without specifying further circumstances, to mean that an individual had had a live-born child that had then passed away, and included these responses to mean that an individual was a parent. We excluded questions where responses indicated that the individual had either aborted or miscarried a pregnancy, or that the individual was pregnant at the time of questioning but with no confirmation since as to whether that child had been born. Based on official statistics indicating that approximately 15% of households include non-dependent children (42% dependent, 43% none) ^44^, we also excluded any responses which indicated that the individual lived with children, but did not confirm whether the individual was responsible for that child.

**Being a carer** was defined as looking after or giving help or support to someone because of long-term physical or mental health conditions/illnesses or problems, and not part of paid employment ^45, 46^. The most common situation is caring for a parent or grandparent, but can also include own children or other family members ^25^. Caring was captured by the same question at six different age-years: “Are you currently a full/part-time carer?”, with yes/no responses.

Although current R scripts are set to define social roles as described above, to enable flexibility for future research, our available list of root questions and auxiliary variables is broader, for example, including questions detailing part-time education, part-time working, irregular/gig work, having a girlfriend or boyfriend (regardless of whether living with them), getting engaged to be married, pregnancy (regardless of outcome). These variables are all listed in Extended Data File 1. Instructions on how to update R scripts to vary the definitions of social roles are provided in Extended Data File 2, Box A. The decisions taken and assumptions made that have been described above are summarised in Extended Data File 2 Tables B and C ^33^, including instructions on how data users can reverse these decisions if needed.

### Deriving annual indicators

After exclusions, we worked with 281 root and 130 auxiliary variables. We passed data on the 15,601 G1 participants through four algorithms (‘current’, ‘date’, ‘since’, and ‘age’), each one relating to a category of root questions capturing a social role, to update annual indicators, resulting in up to 15 indicators (one for each age from 16 to 31) per social role where possible (minimum ages ranged from 16 for leaving the parental home and parenthood to 22 for being a carer – specified under ‘Definitions of social roles’: 115 indicators in total across the six roles, and two sub-roles of higher education and full-time employment).

These binary indicators were coded as a 1 if the individual was confirmed to be in a particular role at a particular age-point. For questions in the ‘current’, ‘date’, and ‘since’ categories, some responses also indicated that the individual was *not* in that role, where the indicator would be coded as a 0 (e.g. at age 21 survey ‘Are you a parent?’, for response ‘No’, parenthood indicator at age 21 = 0). For indicators where one question suggested the individual was in that role, and another question suggested not, the indicator was coded as a 1. When being in/out of that role could not be confirmed, the indicator was coded as missing. It was possible to derive a binary value for at least one annual indicator for each social role on 32–47% of the 14,901 original participants (education: n=5358 [4818 specifically higher education]; employment: 7033 [6266 specifically full-time]; leaving parental home: 6282; cohabiting: 6114; parenthood: 6923, carer: 6332). Of the 11–16 possible annual indicators by age 31 (depending on role), the average number of binary values (before imputing missing values) were around three per role, with a slightly higher average for employment and parenthood (education: median=3 [interquartile range 1 to 5; same for specifically higher education]; employment: 5 [3 to 7], specifically full-time: 3 [2 to 4]; leaving parental home: 3 [2 to 5]; cohabiting: 3 [2 to 4]; parenthood: 5 [3 to 7], carer: 3 [2 to 5]).

Further details about question variables belonging to each algorithm category, possible responses, and other associated variables (e.g. dates of events) are provided in Extended Data File 1. R scripts to run the algorithms themselves are provided on https://github.com/pachucasunrise/ALSPAC_social_roles.

For two of the four algorithm categories (‘current’, ‘date’), birth dates of the G1s helped to inform the ages at which different roles occurred: the month and year of birth were available from the mother’s (G0’s) survey at the first ALSPAC visit after the child (G1) was born, and we approximated the birth date as the 15 ^th^ day of the month.

The algorithm categories were:

**1) ‘current’ questions**, capturing whether an individual was in/out of a specific role at the time of the survey/clinic at which the question was asked.

     This was the only set of prospective questions, the remaining algorithm categories covered retrospective questions. If further relevant information from follow-up questions was available, this was used to estimate the age at which they transitioned to that role (e.g. at the age 19 survey, ‘Are you a parent?’, followed by ‘ **If yes**, when did you become a parent’ – ALSPAC variables ccxf4000 [yes/no], ccxf4001 [month of birth], ccxf4002 [year of birth]). If further information about specific dates of roles was not available, the age at which the respondent completed the questionnaire was used (noting that this could be a year or two either side of the intended sweep age); if the age at which the respondent completed the questionnaire was not available, this age was approximated from the date on which the survey was completed (which was always available).

**2) ‘date’ questions**, where the individual gave specific dates (at least the year, sometimes also the month) at which a person was either in or out a role e.g. dates of periods of (un)employment.

     Ages of the individual at these dates were estimated from these date variables and the individuals’ approximate birth dates.

**3) ‘since’ questions**, which asked retrospectively whether an event had occurred since a certain age.

     As it was important to determine the approximate age at which the event occurred, we only used information from questions where the period since age X and the age at which they responded was no more than two years (e.g. at the age 22 survey, ‘Have any of these occurred since you were **21 years of age** and did they affect you?’). Based on this rule, it was possible for a subset of individuals’ responses to be included regarding a particular question (in the example given, the age at which an individual responded ranged from 21.9–24.6 years old – here we only used responses for those we responded by age 23). A positive response was assumed to indicate that the individual was in the associated role at the truncated mid-point between age X and the age when responding (e.g. truncated[21+(23-21)/2]=22 years old).

     Questions covering a period greater than two years were not used to inform annual indicators, but were used to inform descriptive statistics regarding whether an individual had experienced a role at all by ages 25 and 30 (see ‘Composite indicators’ and ‘Data validation’ section)..

**4) ‘age’ questions**, which asked the individual the age at which a particular event occurred.

     Responses to these questions were directly used to update indicators for those ages.

### Imputation of missing data values 

We handled missing indicator values using multiple imputation; the data both before and after imputation are available to request from the ALSPAC team (see ‘Data and Software Availability’).

### MICT method for imputing categorical sequence data

We imputed data on the sequential indicators themselves (e.g. entered employment at ages 16, 17, 18…), using methods originally developed by Halpin (MICT: Multiple Imputation of Categorical Time Series) ^47^. In this method, for each of multiple imputations, gaps in sequences are imputed starting at the edges of the gaps, with the adjacent non-missing indicators informing the imputation. For example, in the current project, if it was unknown whether an individual was or wasn’t in employment at ages 20, 21, and 22, the indicators at age 20 and 22 would be imputed based on values at ages 19 and 23, and thereafter, the indicator at age 21 would be imputed based on the recently imputed values at ages 20 and 22. Although the imputation method uses information from adjacent time-points within individuals, it also draws on the full dataset, leveraging patterns across individuals with similar observed sequences. This allows one to ‘borrow strength’ from the broader sample, improving the plausibility of imputed values even when individual-level data are sparse. This approach is consistent with recommended practice for imputing longitudinal data ^48, 49^, and has been shown to provide stable and reasonable imputations, even without including auxiliary variables.

We implemented the MICT algorithm using the Stata package ‘mict’ ^50^. We carried out 50 imputations, and imputed data separately for men and women, as patterns of missingness are likely to differ by sex ^6, 10, 51^. The models included the following auxiliary variables which were all indicators of familial socio-economic status and could be used to predict missing indicator values alongside the other non-missing indicators themselves: mother’s marital status and mother’s smoking status during pregnancy; parity, mother’s age, and birthweight at time of G1’s birth, mother’s Edinburgh Postnatal depression score, maternal social class, and paternal social class when G1 8 weeks old. Statistics were pooled across imputed datasets using Rubin’s rules ^52^. It is only possible for the MICT algorithms to act on sequences that have at least one non-missing value. Therefore, this could be applied to the 6098–7014 participants who had at least one non-missing annual indicator for the social role in question.

### Checking imputation using standard Multiple Imputation with Chained Equations (MICE)

The MICT algorithms are less established than standard multiple imputation with chained equations (MICE) within the literature, though have the advantage of providing values for indicators at individual age-years. Therefore, as a sensitivity check, we derived composite binary variables with which to implement standard MICE: that is whether an individual had entered each of the six key roles, respectively, by age 30, and the ages at which each first occurred. We calculated descriptive statistics on these standard MICE-imputed data (proportions entering/exiting roles and median ages at which entry/exit occurred) and compared them to the same statistics calculated using the MICT-imputed individual annual indicators. We implemented standard MICE using the ‘mice’ R package, with similar inputs as above (50 imputations, separately by sex, same set of auxiliary variables as above, statistics pooled using Rubin’s rules).

Unlike the MICT method, which imputed individual annual indicators, standard MICE imputed missing values from composite binary variables summarising the individual’s indicator trajectory. At least one positive response that an individual was in a role at any time-point was sufficient for a binary composite indicator of entering a role by age 30 to equal 1. However, a composite indicator equal to 0 would require the individual to confirm that they were not in that role at every time-point. Therefore, there were few 0 values for composite indicators, however, MICE requires to at least have some observed 0 values to impute binary variables. We therefore took a pragmatic approach where we assumed the values for composite indicators of entering a role by age 30 was =0 if the individual was still active in the study at age 30 (had responded to either ‘YPJ’ or covid-19 questionnaires), at least one-third of annual indicators for that role were non-missing, and none of these indicators were =1. In the case of cohabitation and leaving parents, this approach did not produce any 0’s as it was rare for a participant to have at least 33% non-missing annual indicators. Therefore, we did not impute missing values for the composite binary variables summarising cohabitation or leaving parents trajectories.

Imputing whether an individual exited a role before 30 was conditional on an individual having entered that role in the first place. Similarly, imputing an age of entry or exit was conditional on an individual having entered or exited at all. Therefore, we primarily imputed whether the individual had entered each role by age 30, and used passive imputation to derive: a) the age of first entry into a social role (conditional on having an imputed positive role status by age 30); b) the binary indicator of exiting a role by age 30 (conditional on having an imputed positive role status by age 30); c) age of first exit (conditional on having an imputed positive role status by age 30, and restricting the age of exit to occur after age of entry).

## Data validation

Statistics from derived social role indicators in ALSPAC G1s were compared with official statistics and previously published work after imputing missing values. Official statistics data are relatively complete ^53– 55^, and in previously published work, MICE had also been carried out, and published statistics reweighted for non-response and attrition ^10, 16^.

Table 2 presents descriptive statistics on the composite indicators of role entry and exit by age 30, where missing data have been imputed using the MICT method of imputing missing values of individual annual role indicators (these statistics on data prior to imputation are provided in Extended Data File 2, Table D). When we imputed the sequential indicators using MICE, descriptive statistics of the composites of these sequential indicators were very similar to those presented in Table 2 (Extended Data File 2, Table E).

**Table 2.** Descriptive statistics of social role indicators in ALSPAC G1s: entering and exiting roles by age 30 *.

	Men					
Role	% who have entered this role at least once by age 30	Median age of first transition into role **	(IQR)	Of those who entered a role, % who have exited this role at least once	Median duration ***	(IQR)
Further/higher education	18	20	(18 to 22)	6	4	(3 to 7)
Higher education	18	19	(18 to 22)	2	4	(2 to 6)
Employment (any)	35	21	(18 to 22)	11	3	(1 to 6)
Full-time employment	29	22	(20 to 26)	10	2	(1 to 4)
Left parental home	25	25	(22 to 26)	16	2	(1 to 4)
Cohabiting	27	26	(25 to 27)	34	2	(1 to 3)
Parenthood	8	27	(26 to 28)	11	1	(1 to 1)
Carer	3	25	(23 to 28)	73	2	(1 to 3)
	**Women**					
Further/higher education	33	19	(18 to 21)	6	4	(3 to 8)
Higher education	30	19	(18 to 22)	3	4	(3 to 6)
Employment (any)	57	20	(17 to 22)	14	3	(2 to 6)
Full-time Employment	47	22	(21 to 25)	17	3	(1 to 5)
Left parental home	47	23	(22 to 26)	16	3	(1 to 4)
Cohabiting	50	25	(24 to 26)	33	2	(1 to 3)
Parenthood	20	27	(24 to 28)	8	1	(1 to 2)
Carer	6	26	(23 to 29)	58	2	(1 to 3)

*Pooled across 50 imputed datasets. Missing values were imputed using the MICT method, except in the case of higher education where this method was not possible (in this case we used MICE on the composite binary variables of having entered and exited the role by age 30) ^47, 60, 61^. **If transitioned to that role by age 30 ***If both transitioned and exited from a role before age 30

We treated the total number of ALSPAC participants (i.e. 14,901) as the denominator for calculating percentages of those entering a role at least once by age 30, nothing that across roles, for some, there was at least one observed indicator for 4818 to 7033 participants (more detail is provided under ‘Deriving annual indicators’). We expect the percentages to be under-estimates, providing a minimum prevalence; we do not use the latter values as the denominators, as we expect the questions to be far more sensitive to confirming that an individual is in a role, than not, and therefore doing so would produce over-estimates. Indeed, Extended Data File 2, Table D (column ‘% of cohort to % of N) shows that if we did do this many percentages would be extremely high at 73–98%). We do not expect other descriptive statistics, for example, median age of first entering a role, or exiting a role after entry, to be affected by this issue.

When considering percentages of first entering a role by age 30 to be under-estimates, statistics matched well externally reported similar statistics for UK young adults during 2014–2022 (when the ALSPAC G1s would have been aged 22–32) based on data from the EU Labour Force Survey, for four of the six roles ( Table 2) ^55, 56^. For example, 18% of ALSPAC G1 men and 33% of women indicated that they had been in further or higher education by age 30, compared with an estimated 28% of UK young adults being exclusively in education at age 20–24 in 2014–2022. These estimates are further supported by ONS data suggesting that 33% of UK 18-year-olds attended a UK Higher Education institution in 2019 ^57^. The median age of entering further and higher education was 20 and 19, with a median duration before exit of 4 years, corresponding well with the Eurostat mean age of leaving tertiary education at 23 years, and plausible given that most UK undergraduate degrees are 3–4 years long, and students may stay at university to carry out postgraduate studies. Proportions and median ages of those entering roles by age 30, also corresponded well for similar statistics relating to leaving the parental home, and those cohabiting in Eurostat and research carried out in UK Household Longitudinal Study (UKHLS) data for individuals born in 1987–1992 (in the Eurostat report this was defined as ‘consensual union (with/without legal basis)’ ^53, 55, 58^. Statistics on being a young adult carer also matched those reported from UK Household Longitudinal Study (UKHLS) data, where it was estimated that 9% of those aged 16–29 in 2009–2021 were young adult carers (vs. 3–6% in the ALSPAC G1s up to age 31) ^25^. However, the same study reported the median age of starting this young adult caring role was 22–23 years (in ALSPAC this was 25–26, which is likely due to the fact that the earliest time-point that caring status was captured was at age 22, far later than UKHLS).

The exceptions were the roles of employment and parenthood, where prevalence estimates among ALSPAC G1s were far lower than expected ^55, 56^. For example ^57^, among G1 women, 20% had become parents by age 30; UK Office for National Statistics (ONS) data suggest that half of women born in 1990 (around the same time as the ALSPAC G1s) had had children by age 30 ^59^. However, the average ages of first entering employment and parenthood in among these under-captured cases still correspond well to national statistics and research in UKHLS ^58^. The mean age of motherhood was 28 (data not shown, medians reported in Table 2), ONS data suggests it has consistently been 29 for the last few decades – it is likely that the mean age in the ALSPAC G1s is lower as the data currently go up to age 31, whereas ONS data covered women up to age 45) ^53, 54^. Currently, ONS registrations do not collect data on fatherhood.

Few external data sources have reported on exiting of roles; however, 16% of ALSPAC G1s returning to the parental home ( Table 2) was similar to the 13% reported by a recent study of ‘boomerang moves’ in the UK ^16^. When we investigated the 11% of men and 8% of women who at some point said that they were a parent and then later said that they weren’t, this was often accounted for by early step-parenthood. In the study of young adult caring in UKHLS data, approximately half cared for two waves or more (>1 year); in our data, the median duration was 2 years.

Our social role indicators at age 25 and 26 indicate a lower prevalence of each of five of the six roles, when compared with prevalence estimates at age 25–26 in the Next Steps cohort (born at a similar time to the ALSPAC G1s and representative of the UK population; this study did not report on young adult caring) (Extended Data File 2, Table F) ^10, 33^. This is likely to be due to under-capture at individual age years. We find suggestions of this elsewhere, for example, 9% and 19% of 18-year-old G1 men and women were indicated to be in any employment – ONS data suggest this was 44–50% in the same age population in England at the time ^57^. We could not compare descriptive statistics with UK ONS reports on ages of role ‘milestones’ ^53, 54^, as these were reported as earliest ages at which at least 50% of the population were in a role, and it was very rare for 50% of the ALSPAC G1s to be indicated being in a role within a given year..

## Strengths and limitations

We derived annual indicators for the presence of six key social roles across late adolescence to 31 years old in nearly half of all G1 individuals, making full use of all the rich longitudinal information available in the ALSPAC data (including from indirect questions). The annual indicators may allow more complex analyses with more nuanced enquiries than was possible previously, where most studies using these data have relied on direct questioning at one or two specific time-points. Descriptive statistics regarding transitions to further or higher education, leaving the parental home, cohabiting, and being a young adult carer, were in the range of what could be expected, given information from external sources, supporting their use. Although information on employment and parenthood was captured for nearly 5,000 and 3,000 individuals respectively, descriptive statistics indicate that, relative to the other four social roles, they are likely rather under-captured.

The majority of questions for each of the roles were in the form of direction questioning and asking about current role status ( Table 1), with indirect questioning and retrospective questions brought into algorithms to maximise the amount of information to be drawn from the ALSPAC data. The likelihood of recall error of transitioning to any of the six roles is very unlikely, particularly for first transitions to a role, and in the case of exits/re-entering, when there has been a substantial gap of time between exit and re-entry. It is possible that some short periods of not being a role will not have been captured, either due to their perceived temporary nature by the individual (e.g. moving back into the parental home very briefly in between house moves or out of term-time), or because the period has occurred since the most recent time the role was captured by questioning in the ALSPAC survey schedule, but the period also ended before the next time the role was captured. Similarly, a question may have captured only a brief time spent in a role (e.g. seasonal work at full-time hours, out of student term-time) – nonetheless this is still an experience of that role (e.g. full-time working), with the level of stability reflected by subsequent indicators. Many of the questions were not originally designed to capture social roles and so we had to make some assumptions so that these questions could still be used. For example, we assumed that marriage indicated cohabitation, or that if the individual lived with their partner’s parents, they also cohabited with their partner. We made these decisions between under- and over-capture of our defined social roles based on official statistics, that for example, indicated that most married couples do live together. Of the questions indicating continued education (further or higher), no questions distinguished if a person was in further education specifically, only if in either further or higher education, limiting research specifically about further education. As well as indicators of further/higher education, we provide indicators for higher education specifically, to maximise possible use of these data. Similarly, many employment questions indicated that someone was employed but did not specify to what degree – for example part- or full-time, and rarely whether that person was paid. We provide indicators for any employment, and specifically full-time employment, which we can expect to respectively be useful to future researchers.

Timing of these transitions may be subject to error, both because of recall, and given the nature of the information available. Of the four different types of questioning, two are likely to be subject to such error in terms of timing of events. First, the ‘date’ questions, where the participant gave either the month and year, or only the year at which they started and ended certain roles, and so the day was always approximated to the middle of the month (as was the day of their date of birth). However, these questions currently account for only 2% of variables feeding into education indicators, 6% feeding into employment indicators, and no variables feeding into indicators for the remaining four roles ( Table 1), and so the impact of this error on overall descriptive statistics or downstream analyses is likely to be negligible.

Second, the ‘since’ questions, where the participant is asked whether an event has occurred since a certain age year. These questions account for 14–54% of variables feeding into the indicators for the six different roles. In the context of the ‘since’ algorithm, the individual is unlikely to wrongly recall whether the event occurred was before/after the age-year, but given that no further date details are provided, the algorithm approximates this age to be the mid-point of the age period the question covers (from the ‘since’ age point, to the age of the participant at the time of the questionnaire, where possible). As there is a restriction of only applying this rule for periods up to two years, the maximum possible error is one age-year either side of the ‘true’ age. The main impact of these errors is added noise to analyses when exploring relationships between timing of roles and other variables (e.g. determinants or outcomes). Overall patterns of combinations and of timing of roles, and associated cluster analyses are unlikely to be substantially affected. We advise the researchers to bear in mind this possibility for measurement error when using the annual indicators and interpreting specific descriptive statistics of ages at when certain roles occur, for example, particularly when comparing with statistics from more accurate data such as official statistics.

We validated indicators based on two external sources of data: national statistics from ONS and EU Labour Force survey, and a previous research study that provided statistics about a subset of roles at age 25–26, in a cohort born around a similar time to ALSPAC. It must be noted that these sources are representatives of the general population of England and Wales (and in the case of the Labour Force survey, Europe) at the time. However, the population of Avon, from where the ALSPAC cohort was drawn, is known to be relatively slightly more affluent on average that the rest of the UK ^30^. Therefore, we may expect some indicators that are known to be socioeconomically patterned – for example, age of first motherhood - to be different, perhaps slightly later. However, such regional statistics on these roles with which to compare were not available, and we do not expect them to be so different to render the indicators invalidated. Some of the observed sex differences in role entry by age 30 are consistent with known demographic patterns (e.g. earlier union formation and parenthood among women) ^62^, and may also reflect higher attrition among men in ALSPAC. Future users may wish to explore applying weights to account for differential attrition.

On average and for each role, the majority of an individual’s sequence of annual indicators were missing (average numbers of observed indicators ranged from 3 to 5), limiting the use of the annual indicators based on observed data alone. Future users using the annual indicators are advised to implement appropriate multiple imputation of missing values, or to use the indicators to develop composite indicators (as we did for entering /exiting roles by age 30). Confirmation of whether or not an individual was in a particular role was possible for ~5,000 to 7,000 individuals (depending on which role), a higher number than respondents at most sweeps for young adults in ALSPAC. Depending on the research question, developing variables either through using the same algorithms updated for the user’s definitions, or starting with the readily available indicators that we have produced, is likely to provide a better sense of participants’ ‘journeys’ but also confirm a greater number of cases. In many cases, composite variables, derived from these annual indicators (e.g. role entry by age 30), which have been shown to be comparable with official statistics for education, living situation, and being a carer, are likely to be more appropriate than detailed individual-year analysis.

We explored the use of two different imputation methods: the MICT algorithm on the individual annual indicators, and MICE on composite indicators of whether each individual had first entered or exited a role by age 30. The MICT algorithm has been further developed such that there exists a MICT-timing algorithm ^61^, which takes into account the temporality of the missing value, and is likely to impute values even closer to the truth. However, off-the-shelf software packages to implement these new algorithms are rare or couldn’t be used for our – for example, ‘seqimpute’ in R could not incorporate auxiliary variables with which to implement rules about individuals being still active in the study at age 30 ^61^. A similar package in Stata was not available at the time of writing. Future users of the algorithms and annual indicators we have developed should explore if development of such packages have progressed or could be used within their own project.

Finally, although algorithms allow for indicators to denote when an individual is not in a role, and for individuals to transition back out of roles (role ‘exit’), we did not have many external data sources with which to validate how well indicators pick up *not* being in a role, or role exit. The proportion of ALSPAC G1s returning to the parental home, and average duration of young adult carer roles, match closely with statistics reported elsewhere ^16, 25^. Further work is needed to validate transitions back out of higher education, employment, cohabiting, and parenthood. Such work could take advantage of new data linkages being made between ALSPAC and administrative datasets (such as further and higher education participation, employment tax, and child benefit records) ^63^, which could also help to make up the shortfall of cases of employment and parenthood.

## Ethics and consent

Ethical approval for the study was obtained from the ALSPAC Ethics and Law Committee and the Local Research Ethics Committees. The initial approval was from Bristol and Weston Health Authority: E1808 Children of the Nineties: Avon Longitudinal Study of Pregnancy and Childhood (ALSPAC). Approved 28th November 1989; Southmead Health Authority: 49/89 Children of the Nineties – "ALSPAC". Approved 5th April 1990; Frenchay Health Authority: 90/8 Children of the Nineties. Approved 28th June 1990. Approvals for all clinics thereafter (committee, approval number, dates), are listed here: https://www.bristol.ac.uk/media-library/sites/alspac/documents/governance/Research_Ethics_Committee_approval_references.pdf. All self-completion questionnaire content is approved by the ALSPAC Ethics and Law Committee.

During the original ALSPAC recruitment phase, written informed consent was obtained from the parents of participating children after receiving a full explanation of the study. Children were invited to give assent where appropriate. Written consent is obtained for all ALSPAC clinics. In the case of questionnaires, consent is implied by returning a completed postal questionnaire or submitting an online questionnaire. Study members have the right to withdraw their consent for elements of the study or from the study entirely at any time. Full details of the ALSPAC consent procedures are available of the study website.

## Data Availability

ALSPAC data access is through a system of managed open access. The steps below highlight how to apply for access to ALSPAC data, including access to the data described in this data note. 1. Please read the ALSPAC access policy (PDF, 627kB) which describes the process of accessing the data and samples in detail, and outlines the costs associated with doing so. 2. You may also find it useful to browse our fully searchable research proposals database, which lists all research projects that have been approved since April 2011. 3. Please submit your research proposal for consideration by the ALSPAC Executive Committee. You will receive a response within 10 working days to advise you whether your proposal has been approved. If you have any questions about accessing data, please email alspac-data@bristol.ac.uk. The ALSPAC data management plan describes in detail the policy regarding data sharing, which is through a system of managed open access. OSF: Data Note: Social roles (further/higher education, employment, living situation, parenthood, and being a carer) in the G1s of the Avon Longitudinal Study of Parents and Children (ALSPAC), https://doi.org/10.17605/OSF.IO/2KCN7.{Herbert *et al.*, 2024} This project contains the following extended data: Extended Data File 1. An .xls spreadsheet describing all variables identified in ALSPAC that capture instances of further or higher education, employment, living situation, parenthood, and being a carer for G1s up to age 31. Extended Data File 2. A word document containing one supplementary box and five supplementary tables. Data are available under the terms of the Creative Commons Attribution 4.0 International license (CC-BY 4.0).

## Software availability

Source code available from: https://github.com/pachucasunrise/ALSPAC_social_roles

Archived software available from: doi: 10.5281/zenodo.14205310 (Stata and R scripts).

https://ideas.repec.org/c/boc/bocode/s458046.html (Stata package ‘mict’) and https://cran.r-project.org/web/packages/seqimpute/index.html (R package ‘seqimpute’ version 2.1.0).

License: GNU GPL v3

## References

[1] Irwin CE Jr : Young adults are worse off than adolescents. *J Adolesc Health.* 2010;46(5):405–6. 10.1016/j.jadohealth.2010.03.001 20413074

[2] Hargreaves DS Sizmur S Viner RM : Do young and older adults have different health care priorities? Evidence from a national survey of English inpatients. *J Adolesc Health.* 2012;51(5):528–32. 10.1016/j.jadohealth.2012.05.016 23084177

[3] Neinstein LS Irwin CE Jr : Young adults remain worse off than adolescents. *J Adolesc Health.* 2013;53(5):559–61. 10.1016/j.jadohealth.2013.08.014 24138763

[4] Sacker A Cable N : Transitions to adulthood and psychological distress in young adults born 12 years apart: constraints on and resources for development. *Psychol Med.* 2010;40(2):301–13. 10.1017/S0033291709006072 19531275

[5] Lacey RE Sacker A Bell S : Work-family life courses and BMI trajectories in three British birth cohorts. *Int J Obes (Lond).* 2017;41(2):332–339. 10.1038/ijo.2016.197 27811951 PMC5309340

[6] Green MJ Leyland AH Sweeting H : Causal effects of transitions to adult roles on early adult smoking and drinking: evidence from three cohorts. *Soc Sci Med.* 2017;187:193–202. 10.1016/j.socscimed.2017.06.018 28663104 PMC5529289

[7] Schulenberg J O'Malley P Bachman J : How social role transitions from adolescence to adulthood relate to trajectories of well-being and substance use: Institute for Social Research.University of Michigan, Ann Arbor,2004. Reference Source

[8] Schulenberg J Schoon I : The transition to adulthood across time and space: overview of special section. *Longit Life Course Stud.* 2012;3(2):164–172. 10.14301/llcs.v3i2.194 26473017 PMC4603838

[9] Schoon I Chen M Kneale D : Becoming adults in Britain: lifestyles and wellbeing in times of social change. *Longit Life Course Stud.* 2012;3(2):173–189. 10.14301/llcs.v3i2.181

[10] Gagné T Sacker A Schoon I : Changes in patterns of social role combinations at ages 25–26 among those growing up in England between 1996 and 2015–16: evidence from the 1970 British cohort and next steps studies. *J Youth Adolesc.* 2021;50(10):2052–2066. 10.1007/s10964-021-01477-1 34272653 PMC8416831

[11] McMunn A Lacey R Worts D : De-standardization and gender convergence in work–family life courses in Great Britain: a multi-channel sequence analysis. *Adv Life Cour Res.* 2015;26:60–75. 10.1016/j.alcr.2015.06.002

[12] Martin P Schoon I Ross A : Beyond transitions: applying optimal matching analysis to life course research. *Int J Soc Res Method.* 2008;11(3):179–199. 10.1080/13645570701622025

[13] Ross A Schoon I Martin P : Family and nonfamily role configurations in two British cohorts. *J Marriage Fam.* 2009;71(1):1–14. 10.1111/j.1741-3737.2008.00576.x

[14] Brugha T Bebbington P Tennant C : The list of threatening experiences: a subset of 12 life event categories with considerable long-term contextual threat. *Psychol Med.* 1985;15(1):189–94. 10.1017/s003329170002105x 3991833

[15] Hagell A Shah R Viner R : The social determinants of young people’s health.2020.

[16] Wu J Grundy E : 'Boomerang' moves and young adults' mental well-being in the United Kingdom. *Adv Life Course Res.* 2023;56: 100531. 10.1016/j.alcr.2023.100531 38054880

[17] Hagell A Shah R Viner R : How many young people are accruing the assets they need for a healthy transition into adulthood?London,2019. Reference Source

[18] Schoon I : Navigating an uncertain labor market in the UK: the role of structure and agency in the transition from School to Work. *Ann Am Acad Polit Soc Sci.* 2020;688(1):77–92. 10.1177/0002716220905569

[19] Lindley J McIntosh S : The social mobility of home ownership: to what extent have the millennials fared worse?University of Sheffield,2019. Reference Source

[20] Heinrich M : The economic state of millennials in America.Joint Economic Committee Democrats,2018.

[21] Association for Young People's Health: Key data on young people 2019.2019. Reference Source

[22] Local Governments Association: Re-thinking youth participation for the present and next generation: education to employment.2020. Reference Source

[23] Leavey C Eastaugh A Kane M : Generation COVID-19. Building the case to protect young people’s future health.The Health Foundation,2019.

[24] The Eurocare Team: Young Adult Caring in Europe.2024. Reference Source

[25] Di Gessa G Xue B Lacey R : Young adult carers in the UK-new evidence from the UK Household Longitudinal Study. *Int J Environ Res Public Health.* 2022;19(21): 14076. 10.3390/ijerph192114076 36360950 PMC9656039

[26] Social Care Institute for Excellence: Introduction for young carers’ and young adult carers’ breaks.2024; (accessed 15th October 2024). Reference Source

[27] Brimblecombe N Knapp M King D : The high cost of unpaid care by young people: health and economic impacts of providing unpaid care. *BMC Public Health.* 2020;20(1): 1115. 10.1186/s12889-020-09166-7 32753040 PMC7409476

[28] Harris PA Taylor R Minor BL : The REDCap consortium: building an international community of software platform partners. *J Biomed Inform.* 2019;95: 103208. 10.1016/j.jbi.2019.103208 31078660 PMC7254481

[29] Boyd A Golding J Macleod J : Cohort profile: the 'children of the 90s'--the index offspring of the Avon Longitudinal Study of Parents and Children. *Int J Epidemiol.* 2013;42(1):111–27. 10.1093/ije/dys064 22507743 PMC3600618

[30] Fraser A Macdonald-Wallis C Tilling K : Cohort profile: the Avon Longitudinal Study of Parents and Children: ALSPAC mothers cohort. *Int J Epidemiol.* 2013;42(1):97–110. 10.1093/ije/dys066 22507742 PMC3600619

[31] Northstone K Lewcock M Groom A : The Avon Longitudinal Study of Parents and Children (ALSPAC): an update on the enrolled sample of index children in 2019 [version 1; peer review: 2 approved]. *Wellcome Open Res.* 2019;4:51. 10.12688/wellcomeopenres.15132.1 31020050 PMC6464058

[32] Herbert A Howe LD Heron J : Data note: social roles (higher education, employment, living situation, and parenthood) in the G1s of the Avon Longitudinal Study of Parents and Children (ALSPAC): extended data. 2024. https://osf.io/2kcn7/ 10.12688/wellcomeopenres.23282.2 PMC12398677 40895224

[33] Herbert A Schoon I McMunn A : Data note: social role transitions (further/higher education, employment, living situation, and parenthood, and being a carer) in the G1s of the Avon Longitudinal Study of Parents and Children (ALSPAC): extended data. 2024. 10.12688/wellcomeopenres.23282.2 PMC12398677 40895224

[34] Shanahan MJ Porfeli EJ Mortimer JT : Subjective age identity and the transition to adulthood: when do adolescents become adults?On the frontier of adulthood: theory, research, and public policy. Chicago, IL, US: The University of Chicago Press;2005;225–55. 10.7208/chicago/9780226748924.003.0007

[35] Benson JE Elder GH Jr : Young adult identities and their pathways: a developmental and life course model. *Dev Psychol.* 2011;47(6):1646–57. 10.1037/a0023833 21668096 PMC3792649

[36] Office for National Statistics (ONS): Employee earnings in the UK: 2022. 2022; (accessed 15th May 2024). Reference Source

[37] Office for National Statistics (ONS): Published tables on zero-hours contract trends. 2024.

[38] UG Department for Education: Participation of young people in education, employment or training. Statutory guidance for local authorities. 2024. Reference Source

[39] Hall SS Zygmunt E : “I hate it here”: mental health changes of college students living with parents during the COVID-19 quarantine. *Emerg Adulthood.* 2021;9(5):449–61. 10.1177/21676968211000494

[40] Wood RG Avellar S Goesling B : The effects of marriage on health: a synthesis of recent research evidence.Nova Science New York, NY,2009. Reference Source

[41] Kansky J : What’s love got to do with it? Romantic relationships and well-being. *Handbook of well-being.* 2018;1–24. Reference Source

[42] Office for National Statistics (ONS): People's living arrangements in England and Wales: census 2021. 2023; (accessed 15th April 2024). Reference Source

[43] Hagger MS Hamilton K : Health behavior, health promotion, and the transition to parenthood: insights from research in health psychology and behavior change.In: Taubman – Ben-Ari O, ed. *Pathways and Barriers to Parenthood: Existential Concerns Regarding Fertility, Pregnancy, and Early Parenthood.*Cham: Springer International Publishing;2019;251–69. 10.1007/978-3-030-24864-2_15

[44] Office for National Statistics (ONS): Families and households in the UK: 2022. 2023; (accessed 15th April 2024). Reference Source

[45] Unpaid care harmonised standard. 2020. Reference Source

[46] Carers Trust: About young adult carers. 2024; (accessed 11th November 2024). Reference Source

[47] Halpin B : Multiple imputation for life-course sequence data. 2012; (accessed 15th April 2024). Reference Source

[48] Huque MH Carlin JB Simpson JA : A comparison of multiple imputation methods for missing data in longitudinal studies. *BMC Med Res Methodol.* 2018;18(1): 168. 10.1186/s12874-018-0615-6 30541455 PMC6292063

[49] Jahangiri M Kazemnejad A Goldfeld KS : A wide range of missing imputation approaches in longitudinal data: a simulation study and real data analysis. *BMC Med Res Methodol.* 2023;23(1): 161. 10.1186/s12874-023-01968-8 37415114 PMC10327316

[50] Halpin B : Multiple imputation for categorical time series. *Stata J.* 2016;16(3):590–612. 10.1177/1536867X1601600303

[51] Zhang J Dashti SG Carlin JB : Should multiple imputation be stratified by exposure group when estimating causal effects via outcome regression in observational studies? *BMC Med Res Methodol.* 2023;23(1): 42. 10.1186/s12874-023-01843-6 36797679 PMC9933305

[52] Rubin DB : Multiple imputation for nonresponse in surveys. New York; Chichester: Wiley;1987. 10.1002/9780470316696

[53] Office for National Statistics: Milestones: journeying into adulthood. 2019. Reference Source

[54] Office for National Statistics (ONS): Milestones: journeying through modern life. 2024; (accessed 15th April 2024. Reference Source

[55] Eurostat: Being young in Europe today.2015. Reference Source

[56] Eurostat Data Browser.2023. Reference Source

[57] Office for National Statistics (ONS): Participation in education, training and employment age 16 to 18. Create your own tables. 2023; (accessed 11th November 2024). Reference Source

[58] Karagiannaki E : Transition to adulthood in an intergenerational family context: a cohort and gender analysis based on understanding society. 2024. Reference Source

[59] Office for National Statistics (ONS): Childbearing for women born in different years, England and Wales: 2020. 2020. Reference Source

[60] Halpin B : Imputing sequence data: extensions to initial and terminal gaps, Stata’s *mi* . Limerick: University of Limerick,2013. Reference Source

[61] Emery K Berchtold A Guinchard A : Treating missing data through multiple imputation with the seqimpute package. 2024. Reference Source

[62] Beaujouan E Ní Bhrolcháin M : Cohabitation and marriage in Britain since the 1970s. *Popul Trends.* 2011; (145):31–55. 10.1057/pt.2011.16 21987012

[63] Longitudinal Linkage Collaboration: UK Longitudinal Linkage Collaboration Guidebook.Linkages permitted by each LPS,2024; (accessed 6th November 2024). Reference Source

