# Supplementary material for: Data Note: Social role transitions (further/higher education, employment, living situation, parenthood, and being a carer) in the G1s of the Avon Longitudinal Study of Parents and Children (ALSPAC)
Source: Wellcome Open Res. 2025 Nov 17;9:735. Originally published 2024 Dec 24. [Version 2] doi: 10.12688/wellcomeopenres.23282.2 (PMC12398677; doi:10.12688/wellcomeopenres.23282.2)
Supplement: Peer review history

# Reviewer report: Reviewer response for version 2

**DOI:** 10.21956/wellcomeopenres.27851.r139894

**Published:** 2025-11-20

**Authors:** Jiawei Wu

**Affiliations:** 1 Shanghai University, Shanghai, China

I do not have further comments

Are sufficient details of methods and materials provided to allow replication by others?

Yes

Is the rationale for creating the dataset(s) clearly described?

Yes

Are the datasets clearly presented in a useable and accessible format?

Yes

Are the protocols appropriate and is the work technically sound?

Yes

Reviewer Expertise:

social demography, family sociology, ageing/gerontology, health/well-being

I confirm that I have read this submission and believe that I have an appropriate level of expertise to confirm that it is of an acceptable scientific standard.

# Reviewer report: Reviewer response for version 1

**DOI:** 10.21956/wellcomeopenres.25662.r129393

**Published:** 2025-09-19

**Authors:** Louis-André Vallet

**Affiliations:** 1 CNRS & Sorbonne University, Paris, France

I do like and applaud the general goal of enriching and improving datasets so that other researchers can use and take advantage of rich and complex longitudinal surveys such as the ALSPAC. In this context, the paper is well designed and appropriately written. Unfortunately, I do not think that its precise goal ("to build valid annual indicators at age 16-31 of being in (or out of) six key roles" (see the last lines of the Background section of the Abstract)) can be achieved.

 At the bottom of the first column of page 7, one can read: "Of the 11-16 possible annual indicators by age 31 (depending on role), the average number of binary values (before imputing missing values) were around three per role". So, for each of the six social roles studied, the individual-level data only comprise a MINORITY of OBSERVED data. For each role, the MAJORITY of the possible annual indicators by age 31 are therefore IMPUTED. Even if I am not an expert in the topic of imputation, I do not think it is possible to build a reliable dataset with a majority of imputed annual indicators, on the basis of a minority of really observed annual indicators.

 Some strange aspects of Table 2 reinforce me in this skeptical view. This is especially the case of the large and systematic differences that appear between men and women in the percentage of those who have entered each role at least once by age 30; for instance, as regards cohabiting, this is 50% for women, but only 27% for men; for any employment, this is 57% for women, but only 35% for men, etc.

 As the imputation process was performed separately for men and women (see the bottom of the first column of page 8), I wonder whether these strange differences could only be the result of a too demanding imputation process (imputing a majority of data on the basis of a minority of really observed data) performed separately by sex.

 To conclude, I am not convinced that the final dataset has an intrinsic sufficient quality and my judgment about the approval status is: NOT APPROVED.

 I have a final general comment. Statistical imputation must be performed very carefully and cautiously in the context of individual-level data within longitudinal surveys. The reason is that these individual-level data are generally used in the context of statistical models where some outcomes are examined and “explained” by a set of previous determinants that often include descriptions of the lives and characteristics of the individuals. If these characteristics are measured with error (if an insufficiently controlled imputation process has introduced errors in these variables), that could potentially seriously bias the results of the analysis.

 MINOR POINTS

 Table 1 – The *** note (Across six main roles etc.) on the last line does not appear within the table.

 Page 8 – First column – End of the first paragraph – I cannot understand [21+(23-21)/2]=21 years old. To me, the result is 22, not 21!

 In the same page, the following sentence very clearly presents what I consider to be the serious pitfalls of imputation: “For example, in the current project, if it was unknown whether an individual was or wasn’t in employment at ages 20, 21, and 22, the indicators at age 20 and 22 would be imputed based on values at ages 19 and 23, and thereafter, the indicator at age 21 would be imputed based on the recently imputed values at ages 20 and 22.”

 Page 10 – First column – Third paragraph – Third line – “among G1 women, 29% had become parents by age 30”. Within Table 2, this is 20%, not 29%!

Are sufficient details of methods and materials provided to allow replication by others?

Partly

Is the rationale for creating the dataset(s) clearly described?

Yes

Are the datasets clearly presented in a useable and accessible format?

Partly

Are the protocols appropriate and is the work technically sound?

No

Reviewer Expertise:

Sociology of social stratification and social mobility, Sociology of education, Quantitative sociology, Social statistics, Longitudinal studies, Statistical modelling of categorical data

I confirm that I have read this submission and believe that I have an appropriate level of expertise to state that I do not consider it to be of an acceptable scientific standard, for reasons outlined above.

# Reviewer report: Reviewer response for version 1

**DOI:** 10.21956/wellcomeopenres.25662.r130674

**Published:** 2025-08-30

**Authors:** Shian YIN

**Affiliations:** 1 Department of Health and Social Care Professions, Institution: Birmingham City University (Ringgold ID: 1725), Birmingham, England, UK; 2 Coventry University London, Dagenham, UK

This data note is a really useful piece of work that makes the most of the ALSPAC study to look at how young people move through major life transitions, like leaving education, starting work, moving out of the parental home, forming relationships, becoming parents, or taking on caring responsibilities.

 The authors make a strong case for why this matters: the timing and mix of these roles can have a big impact on health and well-being, yet there hasn't been much contemporary data to capture the full complexity of these transitions. One of the real strengths here is how the team worked creatively with existing ALSPAC questions, many of which weren't designed for this purpose, to build annual indicators that allow us to see these changes across young adulthood. By sharing their coding and scripts openly, they've also made it possible for other researchers to adapt and reuse their approach.

 The methods are carefully set out and show a real effort to deal with the practical challenges of missing data. Using two different imputation techniques and checking results against national statistics adds credibility, and it's reassuring that indicators like education, leaving home, and cohabitation broadly line up with what we know from other sources. The authors are also transparent about the assumptions they made, e.g., treating marriage as a sign of cohabitation, which helps others judge or refine these choices.

 However, there are clear limitations. The data under-capture employment and parenthood, which are central life roles, and this limits how far the findings can be taken at face value. The issue seems to lie with gaps in ALSPAC's questions rather than the methods themselves, but it still means these indicators should be used with caution. The way further and higher education are grouped together, or how approximate ages had to be used when dates weren't available, also means some of the detail is lost. Because the approach relies heavily on imputing missing information, the exact timing of transitions isn't always precise.

 It's also worth keeping in mind that ALSPAC isn't a perfect reflection of the wider UK population. The cohort is more affluent than average and comes from one region, which may shape the timing of things like parenthood. Including young adult caring is a valuable step, but because the data on this only start from age 22, earlier experiences are probably missed. This is particularly significant for groups such as young people who are not in education, employment, or training (NEETs) and care leavers, whose pathways and responsibilities often diverge from their peers much earlier. As a result, the indicators may underestimate or oversimplify the realities faced by some of the most disadvantaged young people.

 Overall, though, this data note does an excellent job of opening up new possibilities for studying young people's life-course pathways. It's best thought of as a flexible and transparent tool for exploring trajectories within ALSPAC, rather than as a source of national prevalence figures. Its real strength is in helping us see patterns and generate new questions, while being upfront about its limits.

Are sufficient details of methods and materials provided to allow replication by others?

Yes

Is the rationale for creating the dataset(s) clearly described?

Yes

Are the datasets clearly presented in a useable and accessible format?

Yes

Are the protocols appropriate and is the work technically sound?

Yes

Reviewer Expertise:

Experiences of adults who were in out-of-home care as children; Care and leaving care; Child and adult social care; Children protection and welfare; Resilience and Youth agency; Life course perspective; Qualitative methods.

I confirm that I have read this submission and believe that I have an appropriate level of expertise to confirm that it is of an acceptable scientific standard.

# Reviewer report: Reviewer response for version 1

**DOI:** 10.21956/wellcomeopenres.25662.r127695

**Published:** 2025-08-01

**Authors:** Jiawei Wu

**Affiliations:** 1 Shanghai University, Shanghai, China

In this data note, the authors drew on data from the Avon Longitudinal Study of Parents and Children (ALSPAC) and built annual indicators of six critical roles (education, employment, parental coresidence, cohabitation, parenthood, and being a carer) from age 16 to 31 for the G1 cohort born in 1991-1993. The dataset compiled will be of great potential  for future researchers working in the transition to adulthood field using methods such as sequence analysis who will be enabled to study inequalities in the socio-economic drivers and consequences of the pathways to adulthood.

 This data note is technically sound and contains a lot of details in the methods section about how the dataset was constructed. I appreciate the authors' effort in imputing some of the missing values using advanced statistical tools. The authors confirmed the validity of their dataset by comparing some of their descriptives with official statistics and/or results reported by other studies using other data collected in the UK. And when these did not match that well, the authors critically analysed the reasons behind the discrepancy. The authors also acknowledged some limitations of this constructed dataset.

 Overall, this dataset is a valuable contribution and will benefit researchers working on topics related to the transition to adulthood.

Are sufficient details of methods and materials provided to allow replication by others?

Yes

Is the rationale for creating the dataset(s) clearly described?

Yes

Are the datasets clearly presented in a useable and accessible format?

Yes

Are the protocols appropriate and is the work technically sound?

Yes

Reviewer Expertise:

social demography, family sociology, ageing/gerontology, health/well-being

I confirm that I have read this submission and believe that I have an appropriate level of expertise to confirm that it is of an acceptable scientific standard.
